# Computer‐Vision Based Gesture‐Metasurface Interaction System for Beam Manipulation and Wireless Communication

**DOI:** 10.1002/advs.202305152

**Published:** 2023-12-03

**Authors:** Yue Teng Chen, Hai Lin Wang, Shi Sun, Zhang Wen Cheng, Yan Kai Zhang, Sen Zheng, Tai Yi Zhang, Hui Feng Ma, Tie Jun Cui

**Affiliations:** ^1^ State Key Laboratory of Millimeter Waves School of Information Science and Engineering Southeast University Nanjing 210096 China; ^2^ Institute of Electromagnetic Space Southeast University Nanjing 210096 China

**Keywords:** computer vision, gesture‐metasurface interaction system, non‐contact, beam manipulation, wireless communication, programmable metasurface

## Abstract

Hand gesture plays an important role in many circumstances, which is one of the most common interactive methods in daily life, especially for disabled people. Human–machine interaction is another popular research topic to realize direct and efficient control, making machines intelligent and maneuverable. Here, a special human–machine interaction system is proposed and namedas computer‐vision (CV) based gesture‐metasurface interaction (GMI) system, which can be used for both direct beam manipulations and real‐time wireless communications. The GMI system first needs to select its working mode according to the gesture command to determine whether to perform beam manipulations or wireless communications, and then validate the permission for further operation by recognizing unlocking gesture to ensure security. Both beam manipulation and wireless communication functions are validated experimentally, which show that the GMI system can not only realize real‐time switching and remote control of different beams through gesture command, but also communicate with a remote computer in real time by translating the gesture language to text message. The proposed non‐contact GMI system has the advantages of good interactivity, high flexibility, and multiple functions, which can find potential applications in community security, gesture‐command smart home, barrier‐free communications, and so on.

## Graphical abstract

A non‐contact computer‐vision‐based gesture‐metasurface interaction (GMI) system is proposed. It can recognize the gesture commands through computer vision, and convert them into electromagnetic signals via a metasurface for real‐time beam manipulation and wireless communication. The proposed non‐contact GMI system exhibits good interactivity, and is promising to be applied in community security, smart home, barrier‐free communications, and so on.

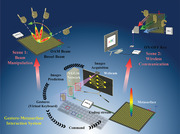

## 1 Introduction

Nowadays, with the rapid development of smart devices, machines with interactive abilities can be well integrated into our lives.^[^ ^1^, ^2^, ^3^, ^4^ ^]^ As one of the most common interactive ways, human hand gestures play an important role in people's daily life. Since gesture recognition is the pivot technology in interactive equipment, there are quite a few methods reported to realize gesture recognition, such as radio frequency (RF) method,^[^ ^5^, ^6^, ^7^, ^8^, ^9^ ^]^ wearable device (WD) method,^[^ ^10^, ^11^, ^12^ ^]^ and computer‐vision (CV) method.^[^ ^13^, ^14^, ^15^, ^16^, ^17^, ^18^ ^]^ RF method can be regarded as a process of RF data collection and analysis by reading the pose of each gesture, and then extracting features through algorithms to finally solve the classification problem. While for the WD method, it usually needs an additional glove or wristband with sensors to obtain external data. Gesture recognition based on the CV method has been proved to be a non‐contact and low‐cost technique, which mainly relies on morphology and classification algorithms. However, to the best of our knowledge, most of the current CV‐based gesture recognition works mainly focus the recognition accuracy improvement, robot control, clinical, and health,^[^ ^13^, ^14^, ^15^, ^16^, ^17^, ^18^, ^19^ ^]^ while there are few reports on CV‐based human–machine interaction to control electromagnetic (EM) fields through gesture recognitions, which limits the further applications of this interactive method.

Metasurfaces, which can be regarded as a two‐dimensional version of metamaterials, have developed quickly in the last decade.^[^ ^20^, ^21^, ^22^ ^]^ Due to the ability to freely manipulate EM waves, metasurfaces have been widely applied in the design of invisible cloaks,^[^ ^23^, ^24^ ^]^ polarization converters,^[^ ^21^, ^25^ ^]^ EM absorbers,^[^ ^26^, ^27^ ^]^ and so on.^[^ ^28^, ^29^ ^]^ In 2014, digital coding and programmable metasurface were first proposed, building a bridge between the digital world and physical world.^[^ ^30^ ^]^ Thereafter, many impressive works have been widely reported based on digital coding and programmable metasurfaces, like space‐time coding metasurfaces,^[^ ^31^, ^32^, ^33^, ^34^ ^]^ reconfigurable intelligent surfaces (RIS),^[^ ^35^, ^36^, ^37^, ^38^, ^39^ ^]^ and artificial intelligent machine.^[^ ^40^ ^]^ Most recently, metasurface‐based human–machine interaction systems have also attracted extensive attentions, such as speech recognition system,^[^ ^41^ ^]^ brain‐computer interaction systems, ^[^ ^42^, ^43^ ^]^ and cyber‐physical robotic system.^[^ ^44^ ^]^ These human–machine interaction systems can recognize commands given by people through voices, brain waves, and postures, and translate them into EM signals, thus achieving real‐time manipulation of EM waves to better serve people.

Hand gestures always play an important role in people's daily communication, especially for disabled people. Here, we propose a CV‐based gesture‐metasurface interaction (GMI) system, which is a special human–machine interaction system. It can recognize gestures and translate them into EM signals for real‐time beam manipulations and wireless communications. A convolutional neural network (CNN) based network Visual Geometry Group 16 (VGG16) is adopted to assist in the training of gesture recognitions, and an element‐individual‐controlled 2‐bit programmable metasurface is meticulously designed for controlling the EM waves. According to the gesture commands, experimental results show that the system can control the metasurface to generate different types of beams in real‐time, such as pencil‐like beam, Bessel beam, and orbital angular momentum (OAM) beam. In addition, it also can implement real‐time wireless communications, by translating the gesture information to EM signals and further sending them to the remote terminal user in form of text message. The proposed system provides a new approach for human–machine interaction to control the EM waves through CV‐based gesture recognition and may find great application potential in community security, gesture‐command smart home, barrier‐free communication, and so on.

## 2 Concept and Design of the GMI System

**Figure 1:**
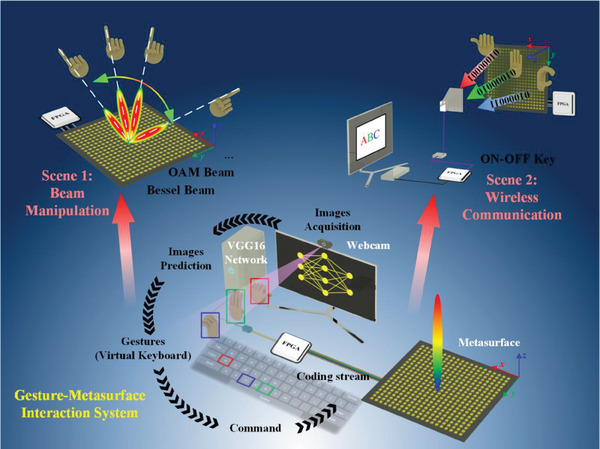
Schematic of GMI system to realize direct beam manipulation and wireless communication. The platform of GMI system involves a webcam, a VGG16‐loaded PC, a FPGA, and a metasurface with a high‐speed control circuit. The real‐time beam manipulation and wireless communication scenes are demonstrated in top left and top right parts, respectively.

The bottom of **Figure** **1** shows the schematic diagram of the transmitting end of the GMI system, which consists of a webcam, a computer, and a 2‐bit programmable information metasurface. When the user puts his hand in front of the webcam, the system will be activated through skin color detection due to the invariance of color skin, then makes a quick snap to obtain gesture images, and displays them on the monitor in real‐time for easy observation and adjustment. The captured gesture images will be further delivered to the pre‐trained VGG16 network, translated them into 8‐bit binary bit stream corresponding to the gesture information, and further sent to the field programmable gate array (FPGA) to drive the programmable metasurface. Finally, a gesture database can be established based on the pre‐trained VGG16 network to mimic the virtual keyboard for information input. In the practical application process, the system first needs to be activated and unlocked, and then the user can select its working scene according to the corresponding gesture commands, such as real‐time beam manipulations or wireless communications in this scheme.

The scene 1 is real‐time beam manipulation, whose conceptual diagram is shown in top left part of Figure 1. Once beam manipulation scene is selected, the user can control the metasurface to generate different types of beams through gesture commands, such as Bessel beam, pencil‐like beam, OAM beam, and so on. In addition, the Bessel beam and pencil‐like beam can be scanned in real‐time with the swing of a finger under the remote sub‐mode, while the topological charge number of OAM beam can be adjusted by using “plus” and “minus” gestures.

The scene 2 is real‐time wireless communication, whose conceptual diagram is shown in top right part of Figure 1. We trained 40 gestures in advance, including alphabets based on American Sign Language (ASL) standard, partially self‐defined punctuations, function keys, and Kaomoji, in which each of them corresponds to only one unique ASCII (American Standard Code for Information Interchange) code number (e.g., A‐01000001, B‐01000010, C‐01000011…). At the transmitting end, the captured gestures are first translated to a series of the corresponding binary bit streams in real‐time according to the training database, and then transmitted by the metasurface using the ON‐OFF Key (OOK) modulation. At the receiving end, the received signal is demodulated into corresponding text content automatically, and then shown in the textbox of the receiving monitor.

## 3 Gesture Training Based on VGG16 Network

**Figure 2:**
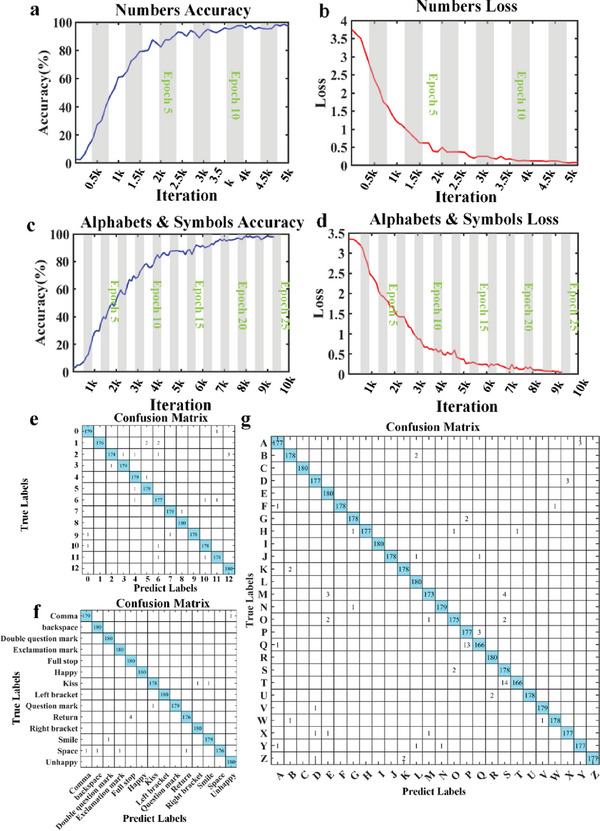
Training performance of VGG16 network in GMI system, in which each label containing 180 validation images. a,b) The accuracy and cross‐entropy loss of the numbers from 0 to 12 for beam manipulation gestures. c,d) The accuracy and cross‐entropy loss of the alphabets and symbols for wireless communication gestures. e–g) The confusion matrices of e) number gestures, f) special symbol gestures, and g) alphabet gestures.

Gesture training is an important part of the GMI system, which is implemented by using VGG16 convolutional neural networks in this scheme,^[^ ^45^, ^46^ ^]^ and three synthetic image datasets of alphabet and some homemade data based on ASL are applied as the training samples.^[^ ^47^, ^48^, ^49^ ^]^ The detailed gesture training framework based on VGG16 network can be seen in Section S1 (Supporting Information). For digital number gestures “0”–“12” used for beam manipulation (for more details see Appendix SI, Supporting Information), named as BM (Beam Manipulation) gestures, ≈5000 iterations and 13 epochs are required to achieve an accuracy of >98% and a cross‐entropy loss close to 0, as shown in **Figure** **2**a,b, respectively. The confusion matrix of number gesture training result is given in Figure 2e to better demonstrate the training performance through 180 verification samples, which shows that all prediction results are in good agreement with the true ones except for almost negligible errors. For 40 different alphabet‐symbol gestures used for wireless communication (see Supporting Information), named as WC (Wireless Communication) gestures, ≈9000 iterations and 24 epochs are required to achieve an accuracy of 98.7% and a cross‐entropy loss close to 0, as shown in Figure 2c,d, respectively. Due to the space limitation, the whole confusion matrix of symbols and alphabets are illustrated in Figure 2f,g respectively. Both of them show good prediction accuracy. It is worth mentioning that pairs “Q‐P” and “T‐S” show relatively larger errors due to the similarity of these two pairs of gestures in morphology, but these errors have little effect on the following experimental results.

## 4 Design of Metasurface

**Figure 3:**
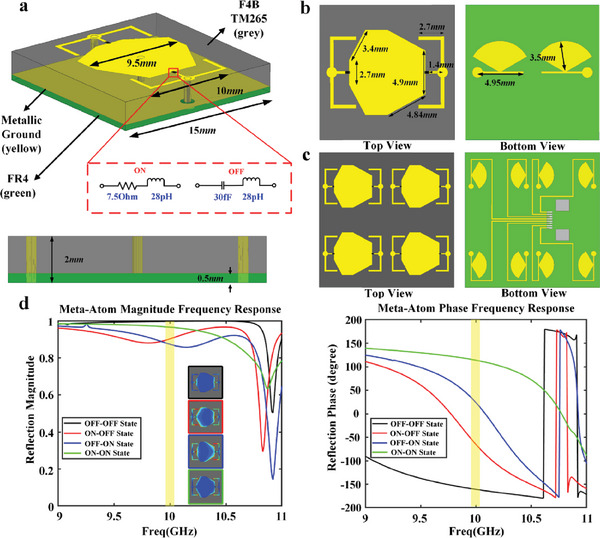
The design of unit element, and its reflection amplitude and phase responses. a) The basic geometric structure and parameters of a single element, mainly includes three metal layers. When the PIN diode is ON (OFF), the equivalent circuit can be regarded as a resistor (capacitor) and an inductor in series. b) Top and bottom views of the meta‐atom. c) Top and bottom views of four cells managed by a shift register. d) Simulated reflection magnitude and phase responses of the element when the PIN diodes are in four different states.

The unit element of metasurface is shown in **Figure** **3**a, which is a three‐metal‐layer structure spaced by a dielectric substrate of F4B (*ε_r_* = 2.65, tan*δ* = 0.001) with a thickness of 1.5 mm and FR4 (*ε_r_* = 4.4, tan*δ* = 0.02) with a thickness of 0.5 mm. The structure on the top layer is composed of an irregular octagonal patch and two E‐shaped arms, with PIN diodes (MADCOM MADP‐000909‐14020x) loaded among them, as shown in Figure 3b. Two fan‐shaped choke capacitors at the bottom layer are connected to two E‐shaped arms on the top layer through metallic holes, respectively, while the irregular octagon on the top layer is connected to the metal ground at the middle layer. The equivalent circuit of the PIN diode is shown in the red dashed box in Figure 3a. When PIN diode is ON/OFF, the equivalent circuit is a cascade of inductor and resistor/capacitor. An 8‐bit shift register is applied to realize independent control of PIN diodes loaded on each unit in every four supercells, which can manage four unit elements at the same time, as illustrated in Figure 3c. The left figure shows structure arrangement on the top layer and the right figure shows an arrangement of the DC bias line with the fan‐shaped choke capacitors at the bottom layer. It should be noted that two grey rectangular patches shown at the bottom layer are the bonding pads of the Flexible Flat Cable (FFC) socket. For more details about the design of control board based on shift registers, see Section S2 (Supporting information). Figure 3d shows the simulated magnitude and phase responses of a single element under the illumination of a linearly polarized incident wave. The results show that the reflection magnitudes are all higher than 0.8 at 10 GHz when two PIN diodes are in OFF‐OFF, ON‐OFF, OFF‐ON, and ON‐ON states, while their reflection phases can be controlled to vary continuously, with a phase interval of 90° achieving 2‐bit phase variation. The simulated electric field intensity distributions of element with the different working states of PIN diodes are demonstrated in the insets of Figure 3d, showing completely different EM responses, which is the fundamental reason for achieving 2‐bit phase responses.

Finally, a programmable information metasurface composed of 20×20 units was designed and fabricated to serve the proposed system, where the PIN diodes loaded on each unit can be individually controlled via bias voltages. For more details about the design of metasurface can be seen in Section S3 (Supporting Information). It is worth mentioning that the feeding horn antenna is placed in front of metasurface at an oblique angle of 30° to avoid the shielding effect of the feeding source on the reflected EM wave, as shown in Figure S4c (Supporting Information). Therefore, the above magnitude and phase responses of a unit element are also investigated based on the case of 30° oblique incidence.

## 5 Experimental Verification

In practical applications, in order to protect personal privacy and system security, the system should be locked when not in use. Hence, an unlocking interface for the system based on the form of Sudoku was created. When the system needs to be used, it must be first unlocked by drawing the correct unlock pattern with the finger. For more details about the design of unlock screen, see Section S4 (Supporting Information).

### 5.1 Real‐Time Beam Manipulation

**Figure 4:**
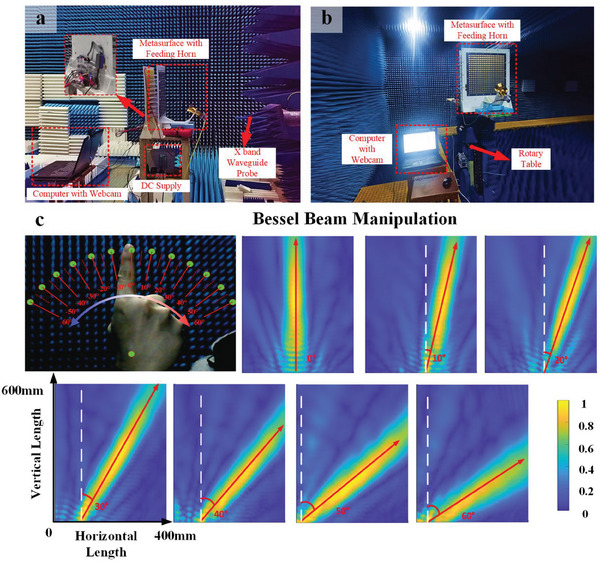
Beam manipulation experimental scenes and setup for GMI system, and the experimental results of Bessel beam manipulation with figure swing. a) Near‐field experimental scene. b) Far‐field experimental scene. c) Experimental results of normalized electric field intensity for gesture‐controlled Bessel beam deflection from 0° to 60° with user interface.

**Figure** **4**a,b illustrates near‐field and far‐field experimental setups of beam manipulation system in the microwave chamber, respectively. A notebook computer and a webcam were used at the control end to store the pre‐trained VGG16 Network of BM gestures and capture the gesture of a user, respectively. Once the gestures were captured by the webcam, they would be recognized and converted to corresponding mode commands according to the pre‐trained VGG16 Network of BM gestures as shown in Appendix SI (Supporting Information), and then the type of beam to be generated will be determined. Next, the required 2‐bit phase distributions of metasurface could be immediately obtained based on the phase compensation theory, which were further converted into corresponding bias voltages for controlling the states of PIN diodes loaded on metasurface through an FPGA module. In the near‐field measurement, an X‐band waveguide probe was located in front of metasurface to detect the electric field distributions of EM waves, as shown in Figure 4a. While in the far‐field measurement, the metasurface was placed on a rotary table and a receiving horn antenna was located in the other side of microwave chamber to receive signals, as shown in Figure 4b.

Five beam modes were implemented to validate the performance of the system for beam manipulation, including pencil‐like beam, dual beam, OAM beam, diffusion beam, and Bessel beam, as shown in Figure S7 in Section S5.1 (Supporting Information). The phase compensation derivations for different beams can be found in Section S7 (Supporting Information). Take the generation and real‐time control of the Bessel beam as an example to show the process and performance of beam manipulation, as shown in Mode 5 of Figure S7a (Supporting Information). A gesture command was sent to the system to make it switch to the beam manipulation mode. Then, a five‐fingered gesture in front of the webcam was shown, so the system can recognize it as number five, and switch to the operation mode of Bessel beam according to the definition of the pre‐trained VGG16 Network of BM gestures. Under this mode, two sub‐modes can be further selected. One was “Auto‐Scanning” mode corresponding to one‐fingered gesture command. In this sub‐mode, the Bessel beam will automatically and periodically scan rapidly within the range of −60° to 60°. The other was “Remote control” mode, corresponding to two‐fingered gesture command. In this sub‐mode, the Bessel beam can be scanned within the range of −60° to 60° with the swing of a finger. Here, the function of “Remote control” mode. The control interface of real‐time beam scanning with finger swing is shown in the up‐left subfigure of Figure 4c. The near‐field measured results of Bessel beam at different deflection angles are given in the other subfigures of Figure 4c, which show that the Bessel beam can be continuously scanned from 0° to 60° in real‐time under the gesture remote control. It is worth mentioning that the measured results from −60° to 0° were not displayed here, which were symmetrical with the results of 0°–60° shown in Figure 4c. The results of other beam modes, such as pencil‐like beam, dual beam, OAM beam, and diffusion beam, can be seen in Figure S8 (Supporting Information).

### 5.2 Real‐Time Wireless Communication

**Figure 5:**
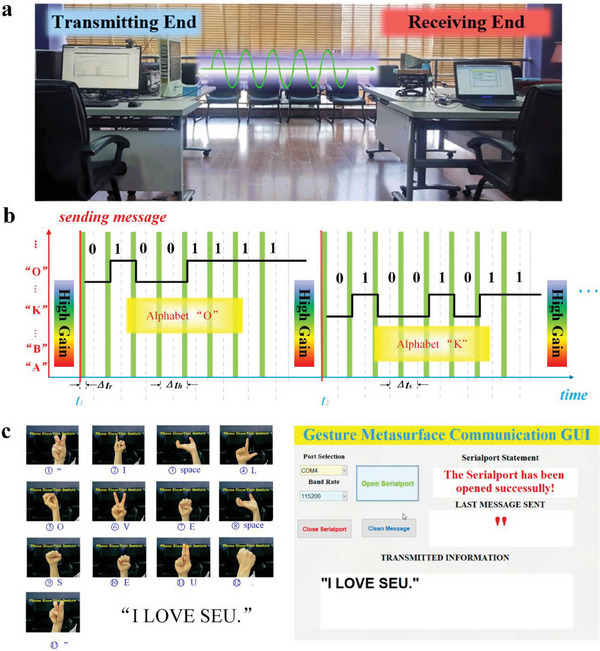
Wireless communication experimental scenes and setup for GMI system, time sequence of metasurface, and partial experimental demonstration. a) Experimental scene, overall setup for the communication system. b) Time sequence of metasurface when “O” and “K” are transmitted, respectively. c) An example “I LOVE SEU.” is demonstrated at the transmitting end and receiving GUI, respectively.

**Figure** **5** demonstrates the experimental setup of a wireless communication system, which consists of a transmitting end and a receiving end. For more details about the workflow and frame of the system, see Figure S9 (Supporting Information). The pre‐trained VGG16 Network of WC gesture that contains 40 different gestures was adopted in this scheme, as shown in Appendix SII (Supporting Information). These gestures can be regarded as a virtual keyboard based on gesture definition, including alphabets, punctuations, carriage return, backspace, and Kaomoji, and it can be further expanded. At the transmitting end, first wireless communication mode was selected and unlocked the system, and then input messages remotely in real‐time using the defined virtual gesture keyboard through a webcam. The captured gesture images were delivered to the pre‐trained VGG16 Network of WC gestures and translated into 8‐bit binary bit streams associated with corresponding RS232 commands. Then, these RS232 commands were further converted into corresponding ASCII codes and sent by the programmable information metasurface using OOK modulation. Take sending the message “OK” as an example, the associated ASCII codes for “O” and “K” were “0 100 1111” and “0 100 1011”, respectively. Figure 5b shows the time‐varying coding sequences of ASCII codes for “O” and “K”, where digital codes “1” and “0” correspond to high gain pattern and low gain pattern of metasurfaces, respectively. It was worth mentioning that the high gain pattern and low gain pattern of metasurfaces were corresponding to the pencil‐like beam and diffusion beam modes of metasurface, respectively, whose measured far‐field radiation patterns can be seen in Figure S8a,b (Supporting Information). More principle and detail of OOK in this scheme were described in Section S8 (Supporting Information). Here, the sampling interval time was *Δt_s_* = 2.048 ms, and the rendering delay of the control board was *Δt_r_* = 25.6 μs marked by green bar in Figure 5b. It was paramount that holding time *Δt_h_* should be much longer than *Δt_r_* to prevent sampling during the circuit board's rendering, which might cause inevitable bit errors. High gain pattern was set as the default state of metasurface when the camera was waiting for commands (gestures), more detail about relative setting can be seen in Section S8 (Supporting Information). A full wireless communication process to send a message “I LOVE SEU.” is demonstrated in Figure 5c, in which the left part was the transmitting end, showing the text message to be transmitted and its corresponding gesture commands. The right part was the receiving end, showing the received text message “I LOVE SEU.” that was displayed on the graphical user interface (GUI) of the receiving end. The results showed that the gesture commands could be correctly recognized and sent to the receiving end in form of a text message via the proposed GMI system, and the detailed real‐time operating process can be seen in Video S1 (Supporting Information). In addition, two more wireless communication demonstrations including “Backspace” and “Carriage Return” functions are given in Videos S2 and S3 (Supporting Information), respectively.

In current version, although the processing data and metasurface response could be finished in millisecond level, the response speed of the system was still not fast enough due to the time cost in gesture capture process, which was set to 3 s in this design. However, the gesture capture time was expected to be greatly reduced through adequate sign language training. For more details about the discussion of time response of the system, see Section S9 (Supporting Information).

## 6 Conclusion

We propose an integrated CV‐based GMI system, which can implement real‐time beam manipulations and wireless communications through gesture commands. A 2‐bit programmable metasurface with the capability of pattern coding in quick response is designed to serve the system, and the deep learning technique is used to combine with metasurface to achieve signal conversion from hand gestures to EM waves. The performance of this system has been experimentally validated, and the results show that the system can be directly controlled by gesture commands of users without any peripherals to realize real‐time beam manipulations and remote wireless communications. In addition, the self‐defined gesture function keys can bring more degrees of freedom to realize further complicated requirements. Further considering the practicality of the system, a two‐hand gesture recognition strategy can be developed in the future work, and more effective modulation methods can be adopted for wireless communication mode, such as frequency‐shift keying (FSK) or quadrature phase shift keying (QPSK). It can be expected that GMI system with stronger interaction capability and more intelligence by involving artificial intelligence (AI) algorithms and some self‐adaptive mechanisms, and is promising to be applied in non‐contact sterile barrier system, gesture‐command smart home, barrier‐free community, and so on.

## Conflict of Interest

The authors declare no conflict of interest.

## Supporting information

Supporting Information

Supplemental Video 1

Supplemental Video 2

Supplemental Video 3

## Data Availability

The data that support the findings of this study are available from the corresponding author upon reasonable request.
