# Supplementary material for: Computer‐Vision Based Gesture‐Metasurface Interaction System for Beam Manipulation and Wireless Communication
Source: Adv Sci (Weinh). 2023 Dec 3;11(5):2305152. doi: 10.1002/advs.202305152 (PMC10837369; doi:10.1002/advs.202305152)
Supplement: Supplementary file 1 — Supporting Information [file ADVS-11-2305152-s001.pdf]

ADVANCED SCIENCE
Open Access

Supporting Information

for *Adv. Sci.*, DOI 10.1002/advs.202305152

Computer-Vision Based Gesture-Metasurface Interaction System for Beam Manipulation and Wireless Communication

Yue Teng Chen, Hai Lin Wang, Shi Sun, Zhang Wen Cheng, Yan Kai Zhang, Sen Zheng, Tai Yi Zhang, Hui Feng Ma and Tie Jun Cui**

Computer-Vision based Gesture-Metasurface Interaction System for Beam Manipulation and Wireless Communication

Yue Teng Chen, Hai Lin Wang, Shi Sun, Zhang Wen Cheng, Yan Kai Zhang, Sen Zheng, Tai Yi Zhang, Hui Feng Ma*, and Tie Jun Cui*

State Key Laboratory of Millimeter Waves, School of Information Science and Engineering, Southeast University, Nanjing 210096, China
Institute of Electromagnetic Space, Southeast University, Nanjing, 210096, China

*Corresponding author. Email: hfma@seu.edu.cn, tjcui@seu.edu.cn.

The Supporting Information includes:

1. Section S1. Framework of VGG16 and training results

2. Section S2. Design of control circuit

3. Section S3. Fabricated prototype of the programmable metasurface

4. Section S4. GMI system Unlock process

5. Section S5. The workflow and experimental results of real-time beam manipulation

6. Section S6. The workflow of wireless communication and experimental setup

7. Section S7. Phase compensation of different-type beams in beam manipulation

8. Section S8. OOK modulation and demodulation in wireless communication

9. Section S9. Response time for the system

10. Appendix SI: Gestures definition for beam manipulation

11. Appendix SII: Gestures definition for wireless communication

Section S1. Framework of VGG16 and training results

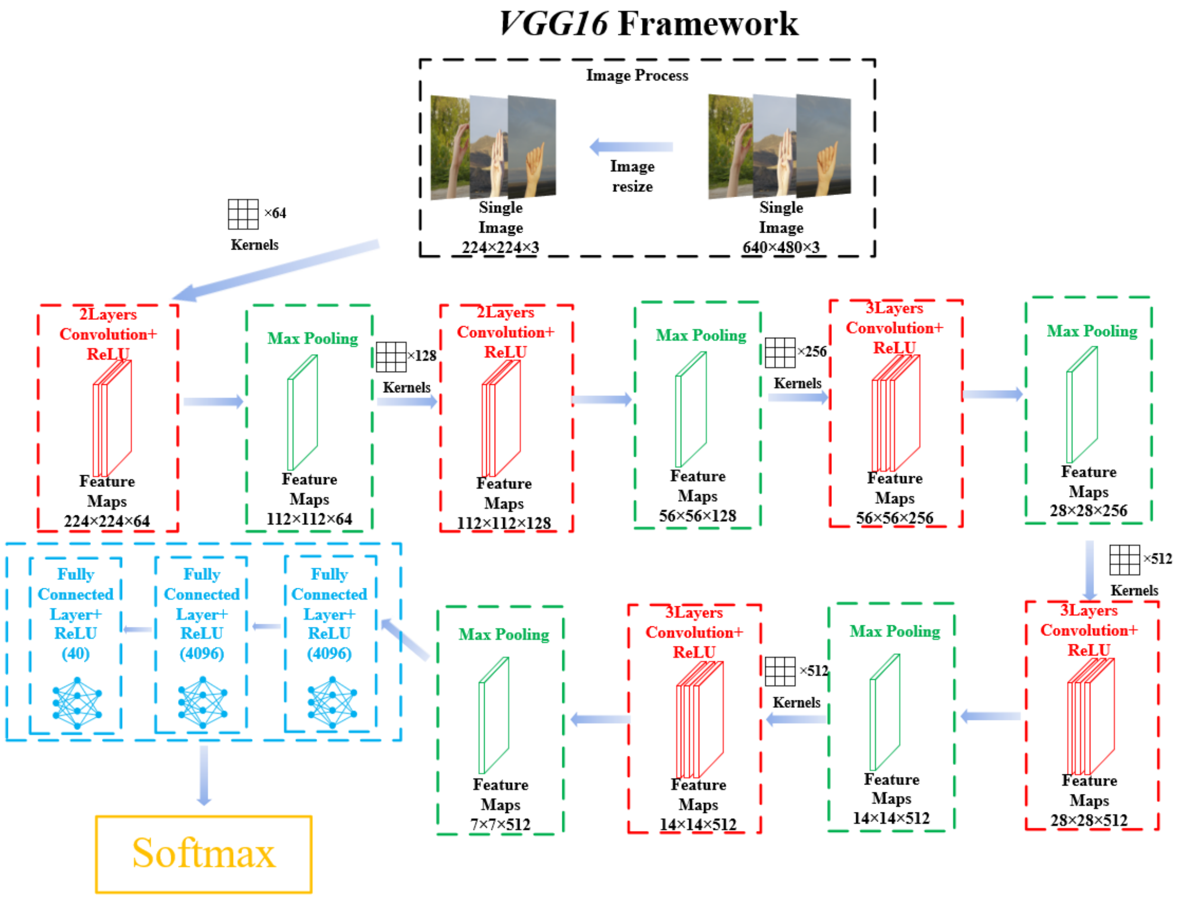

Figure S1. The basic framework of VGG16 in training tasks. Due to the fixed input size, all the input images should be resized to the RGB image with size of 224×224×3.

In the pre-trained stage, hand gestures, including numbers, alphabets, punctuations, kaomoji, and some function keys, are trained by VGG16 network. The training platform is equipped with an Intel i9-13900KF 24-core processor, 64GB RAM, a single slice of NVIDIA Geforce RTX 4090 with 24 GB video memory, and the training process is executed on Windows operation system .The hand gestures and their corresponding meanings can be found in Appendix SI at the end of this Supporting Information, where the alphabets and numbers are defined based on ASL standards, while the others are self-defined.

Figure S1 shows the framework of the VGG16 network, which includes image preprocessing, feature extraction and classification. The images need to be resized to fit the network before inputting, as depicted in the black dashed box in Figure S1. After the preprocessing, feature extraction stage is applied, including several convolution and max pooling layers. As the convolution process proceeds, the size of the image gets smaller but the dimension expands, this stage is marked by the red and green dashed boxes in Figure S1. Finally, three fully connected

layers and a softmax layer are the last step of this network to finish the prediction and classification, which is described by blue and orange dashed box.

Section S2. Design of control circuit

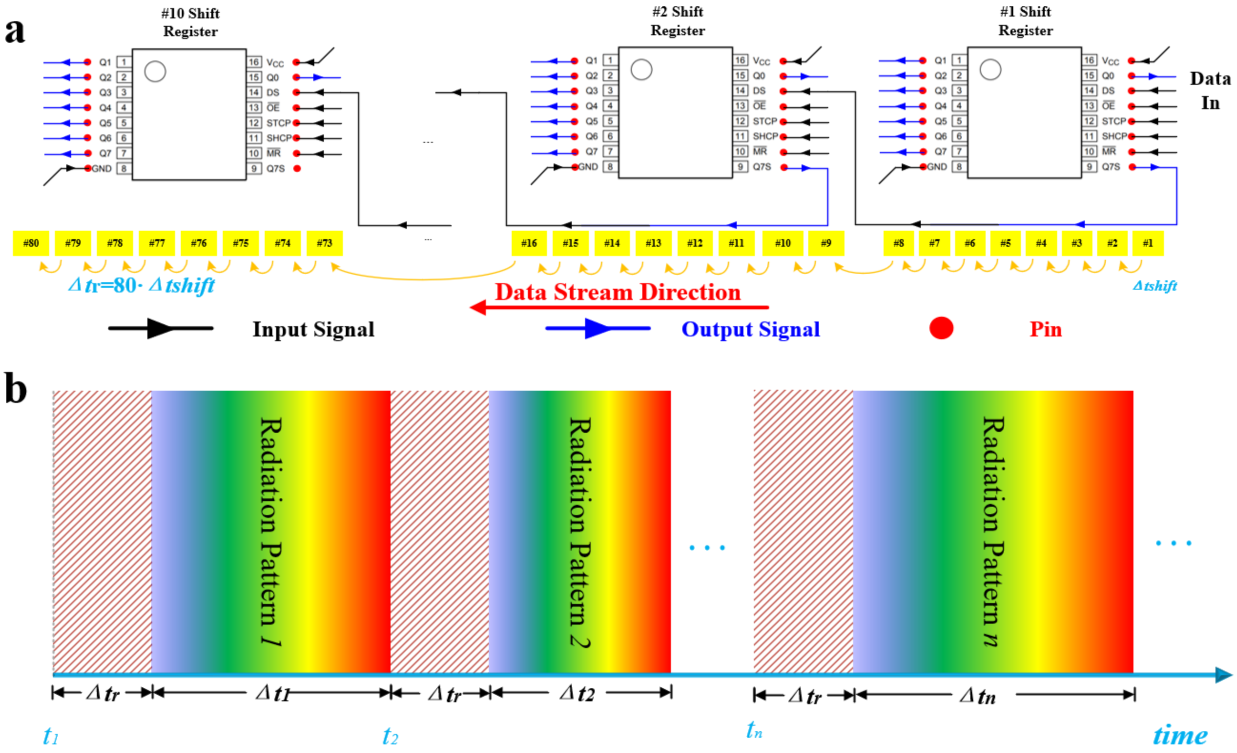

Figure S2. Operation principle of the control circuit and timing analysis. a. Data shifts in a single row. b. Timing analysis of metasurface.

For the programmable metasurface design, the individual control of a 2-bit element is relatively complicated. Here, a register-based circuit board has been used in this design due to its merit of IO port-saving and simple wiring. Figure S2a shows the operation principle of a single row of the designed control circuit board. Each row consists of 10 8-bit shift registers in cascade form due to the capability of the cascade of NXP 74HC595D [S1]. It should be noted that this shift register chip has a maximum shift frequency of 100 MHz, and that is one of the keys to realize quick response of the whole metasurface. A 3.3V direct current (DC) power supply is connected to the Vcc and MR_bar of each chip. The GND port is connected to the negative electrode of the DC power supply. Moreover, STCP and SHCP ports are the release signal and shift signal of each chip, respectively. Q0-Q7 ports are the 8 parallel outputs of each chip, and they are connected to the 8 PIN diodes of a supercell. To achieve cascade form, the most important is to connect the former chip's Q7S port to

the latter's DS port. The data stream is input at the DS port of the first shift register with the overall direction from right to left. It is crucial to note that in this case, the current in each IO port located in our commercial development FPGA is not strong enough to drive so many chips, especially for the "SHCP" and "STCP" signals. At the same time, a high-frequency shift signal consumes more power, it also tests the drive capability of this system. To provide enough current strength, 10 IO ports on FPGA are used and gathered for SHCP and STCP signals, respectively, a total of 30 IO ports are occupied on the FPGA to manipulate the active 2-bit programmable metasurface. Meanwhile, we define the period of SHCP Δt_{shift} is 320 ns, and 10 shift registers in cascade form lead to a complete shift, which is the period of STCP signal (rendering time or refresh time), Δt_r=25.6 us. Accordingly, each row of the control circuit controls 2-row meta-atoms, which means for a 20×20 metasurface, 10 such rows are needed. It is worth noting that the coding streams for 10 rows are input simultaneously, that is to say, the total rendering time equals to Δt_r. Figure S2b illustrates the time sequence of metasurface when it operates. The shadow region represents the metasurface under rendering while the rainbow region represents that the desired radiation pattern is emitted, where the holding time Δt_n is defined by the users.

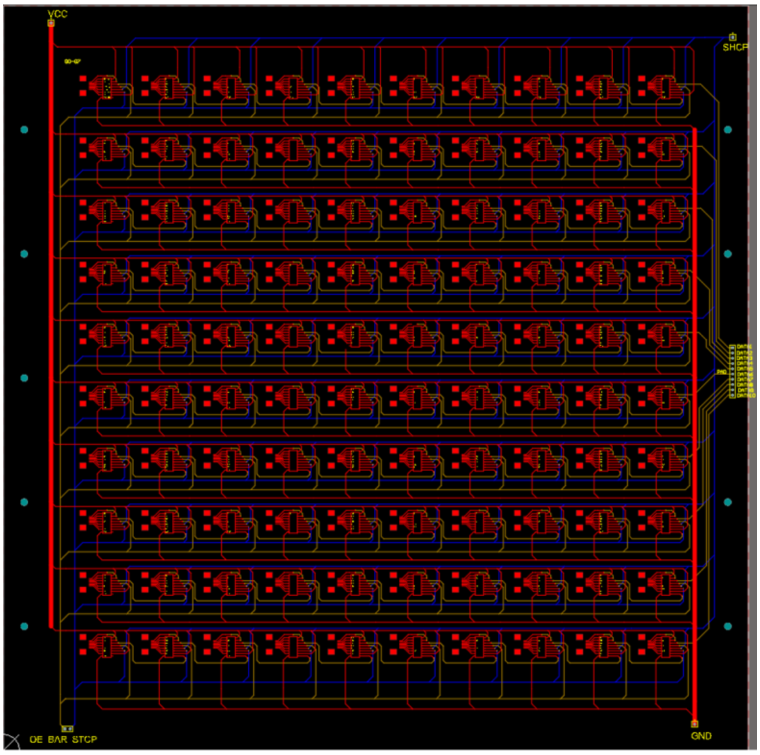

Figure S3. Layout of the control circuit. 10-way coding streams should be input at the right side simultaneously.

The layout of the circuit board is shown in Figure S3. The circuit board is a 3-layer structure

spaced by 2-layer FR4 (h=0.5mm, ε=2.2, δ=0.001) substrates, and 10 data inputs at the right side and other inputs including signal and power are located at four corners.

Section S3. Fabricated prototype of programmable metasurface

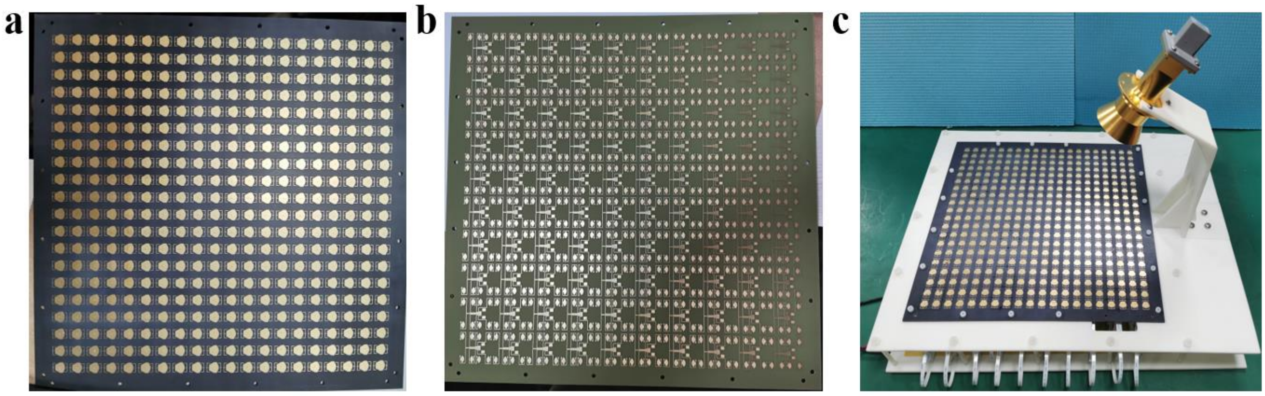

Figure S4. Fabricated metasurface. a. Top view of prototype. b. Bottom view of prototype. c. Overall view of metasurface and control circuit with their connection.

Figures S4a and b show the top and bottom views of the fabricated metasurface, respectively, while the overall view of the metasurface is shown in Figure S4c, which is fed by a horn antenna with a 30° oblique incidence. Metasurface and its control circuit boards are up and down arranged with total of 100 flexible flat cables (FFC) between them.

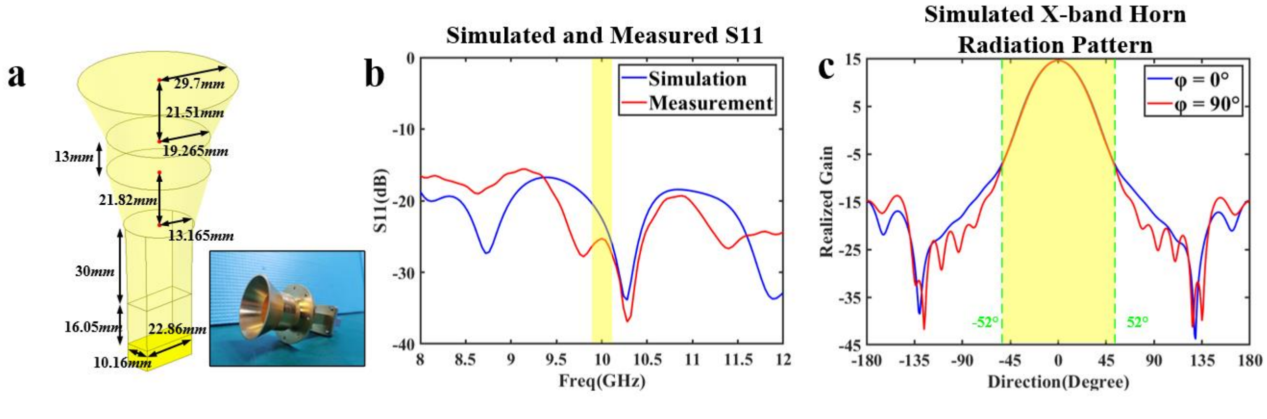

Figure S5. Fabricated X-band horn and its simulated and tested performance. a. The schematic of designed X-band horn and its geometric parameters. b. Simulated and tested reflection coefficient for this designed horn. c. Simulated radiation performance at 10 GHz.

We designed a dual-mode X-band horn as the feeding source of metasurface, as shown in Figure S5a. The simulated and measured results of the reflection coefficient (S11) and far-field radiation patterns are shown in Figures S5b and c, respectively. The results show that S11 is lower

than -15 dB within the whole X band from 8-12 GHz, while the realized gain patterns for both E-plane and H-plane are quite symmetrical with an overlap angle range covering from -52° to 52° at 10 GHz.

Section S4. GMI system Unlock process

For a personally used system, an unlock mechanism is necessary for the GMI system. For this purpose, we established a gesture-unlocking interface, and the unlocking process is shown in Figure S6. When we show our hand in front of a webcam, a welcome interface appears on the screen through the color-skin-based detection, as shown in step 1 of Figure S6. Then, we use the finger to control unlock bar button and slide it from the left to right to activate the system. Further, a gesture-based Sudoku unlock comes out in the central of 9 white-circle regions, as shown in step 2 of Figure S6. Only when we can draw the correct unlock pattern by moving the finger, the system will be finally unlocked and controlled by the user, as shown in steps 3 and 4 of Figure S6. The experimental demonstration of unlock process can be seen at the beginning of Supplementary Video S1 and Video S2.

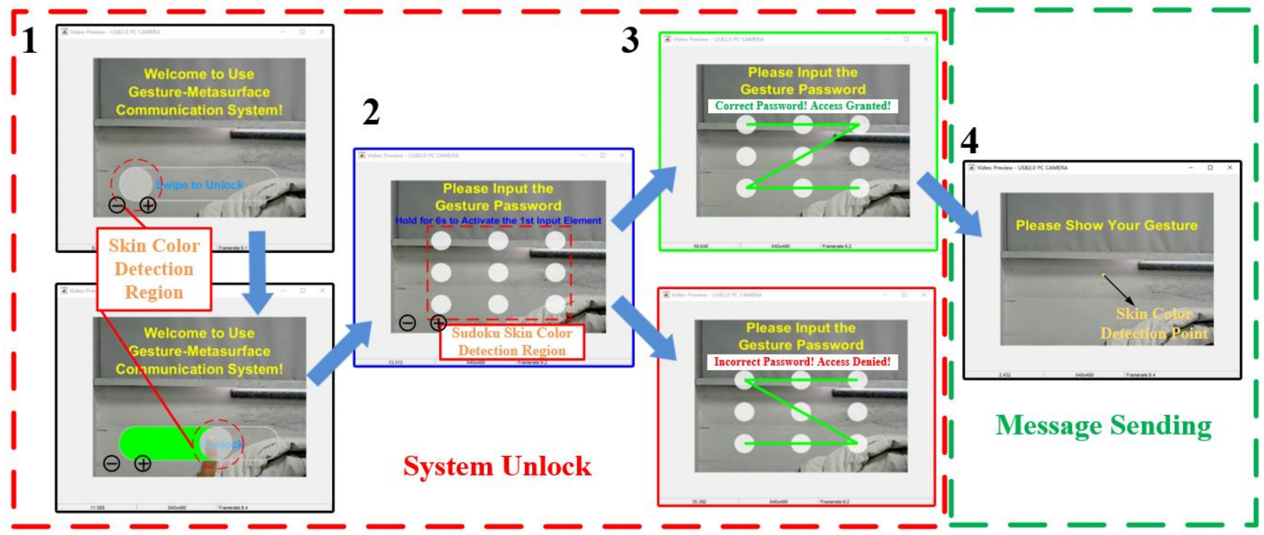

Figure S6. Workflow of unlock the system to realize message sending.

Section S5. The workflow and experimental results of real-time beam manipulation

S5.1. The workflow of beam manipulation

Five functionalities were implemented to demonstrate the real-time beam manipulation ability of

this system, including pencil-like beam, dual beam, OAM beam, diffusion beam and Bessel beam, which correspond to the gesture commands of “1” to “5”, respectively, as shown in Figure S7a. In addition, the pencil-like beam and Bessel beam can be further controlled to remote in real-time by automatically scanning within the range of -60° to 60° if sub-mode 1 is selected, or swinging the finger to control the direction if sub-mode 2 is chosen, and OAM beam can be further controlled to increase or decrease the topological charge in real time by showing “plus” gesture (self-defined gesture “11”) and “minus” gesture (self-defined gesture “12”), respectively, whose topological charge can be continuously controlled from -3 to 3 orders. Figure S7b shows the real-time beam direction control User Interface (UI) for pencil-like beam and Bessel beam, in which the green region is used for color skin detection.

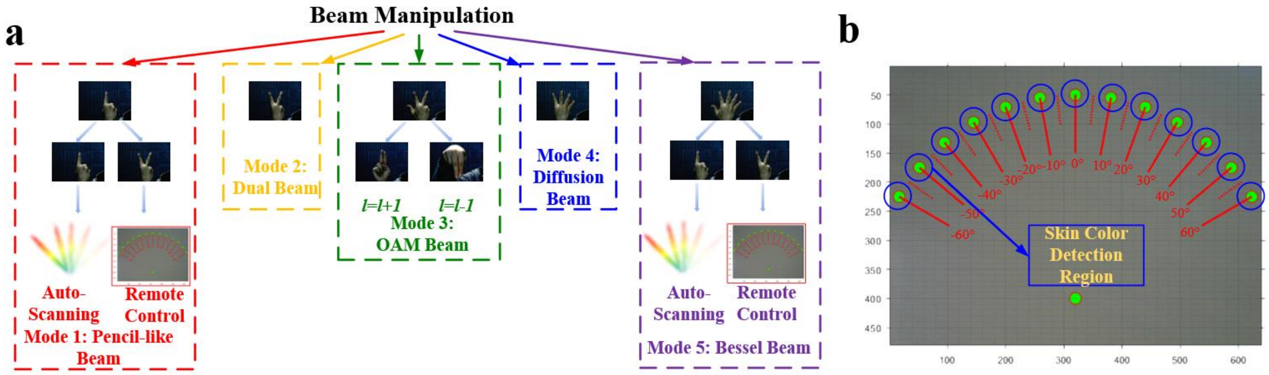

Figure S7. Workflow of Beam Manipulation. a. Different mode choices for beam manipulation. b. Beam direction control UI for pencil beam and Bessel beam.

S5.2. Experimental results for Beam Manipulation

Figure S8a shows the radiation pattern of pencil-like beam deflection under the gesture remote mode. The beam can be controlled to continuously scan within ±60°, with only about -3 dB gain loss at the largest scanning angle. Figure S8b shows the experimentally verified dual-beam and diffusion beam. Two symmetrical radiation beams at ±30° can be achieved in dual-beam mode, while the radiation beam is randomly scattered in the diffusion beam mode. Figure S8c is the measured phase distribution of the designed OAM beam at a distance of 700 mm away from the metasurface, where the topological charge number varies from -3 to +3, respectively.

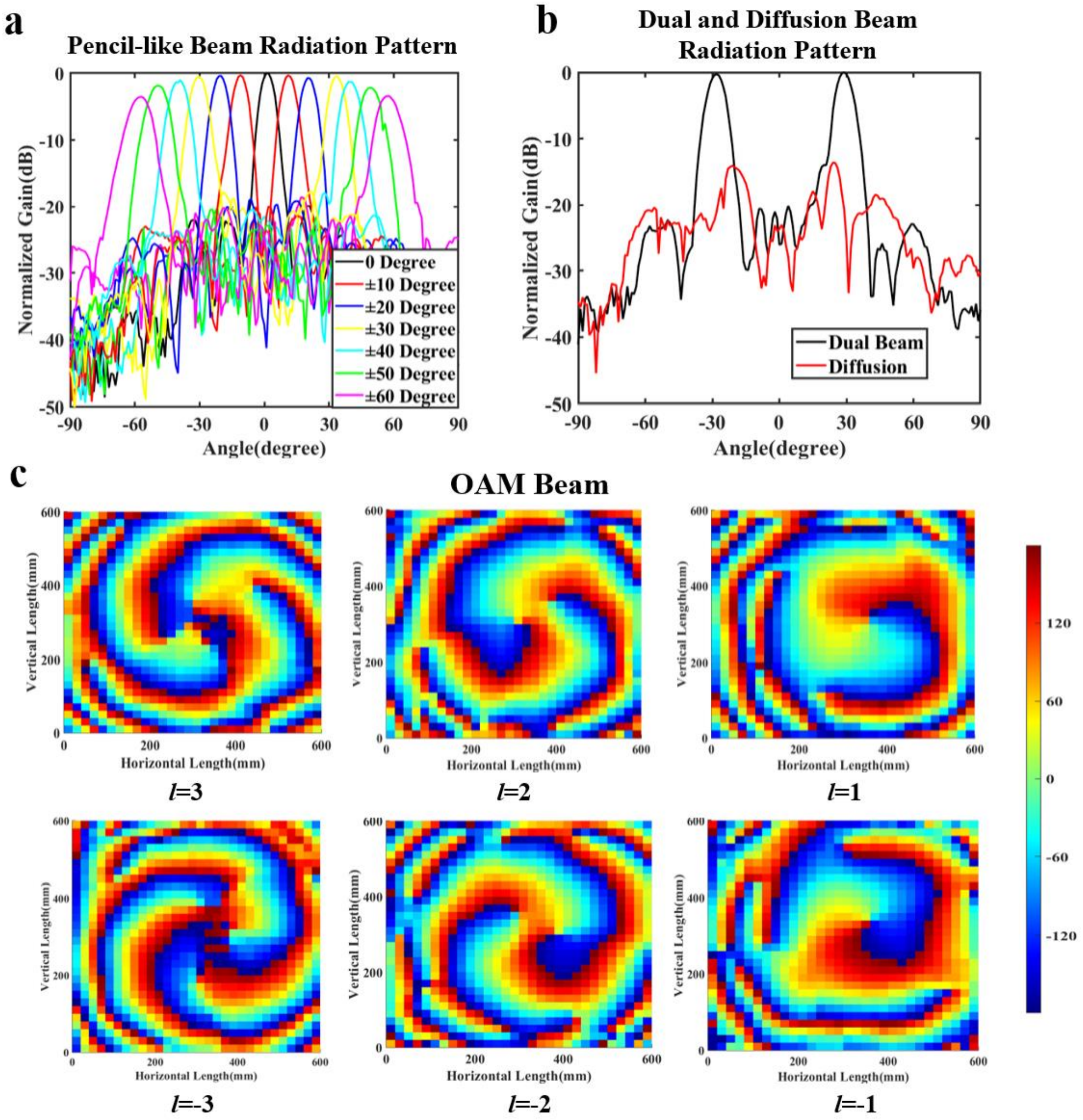

Figure S8. Experimental results for pencil-like beam, dual-beam, diffusion beam and OAM beam. a. Experimental results of pencil-like beam in different direction. b. Experimental results of the dual-beam and diffusion generated by metasurface. c. Experimental near-field phase state of OMA beam in different topological charge number.

Section S6. Workflow of wireless communication and experimental setup

S6.1. Workflow of Wireless Communication

Figure S9a shows the workflow of the wireless communication of the GMI system. At the transmitting end, an input gesture image is first recognized by the webcam and then converted to the corresponding RS232 command, which is further sent to FPGA and translated into a coding stream. After that, the coding stream is delivered to the metasurface to generate the corresponding high-gain pencil-like beam (digital code “1”) and low-gain diffusion beam (digital code “0”),

respectively, which are used as an ON-OFF Key (OOK) modulation. At the receiving end, the modulated signals are received by a horn antenna and decoded by corresponding components, then, the decoded coding sequence is translated to the corresponding text message and displayed at the displayer. As for the GUI design at the receiving displayer, not only the editing-textbox will be displayed, but also a port selection and baud rate are shown in this GUI. An “open” and a “close” buttons are designed to activate and shut down the receiving end with a corresponding hint in “Serial port Statement”. Furthermore, when the editing box is full, the “clean message button” can be used to remove all the information received before.

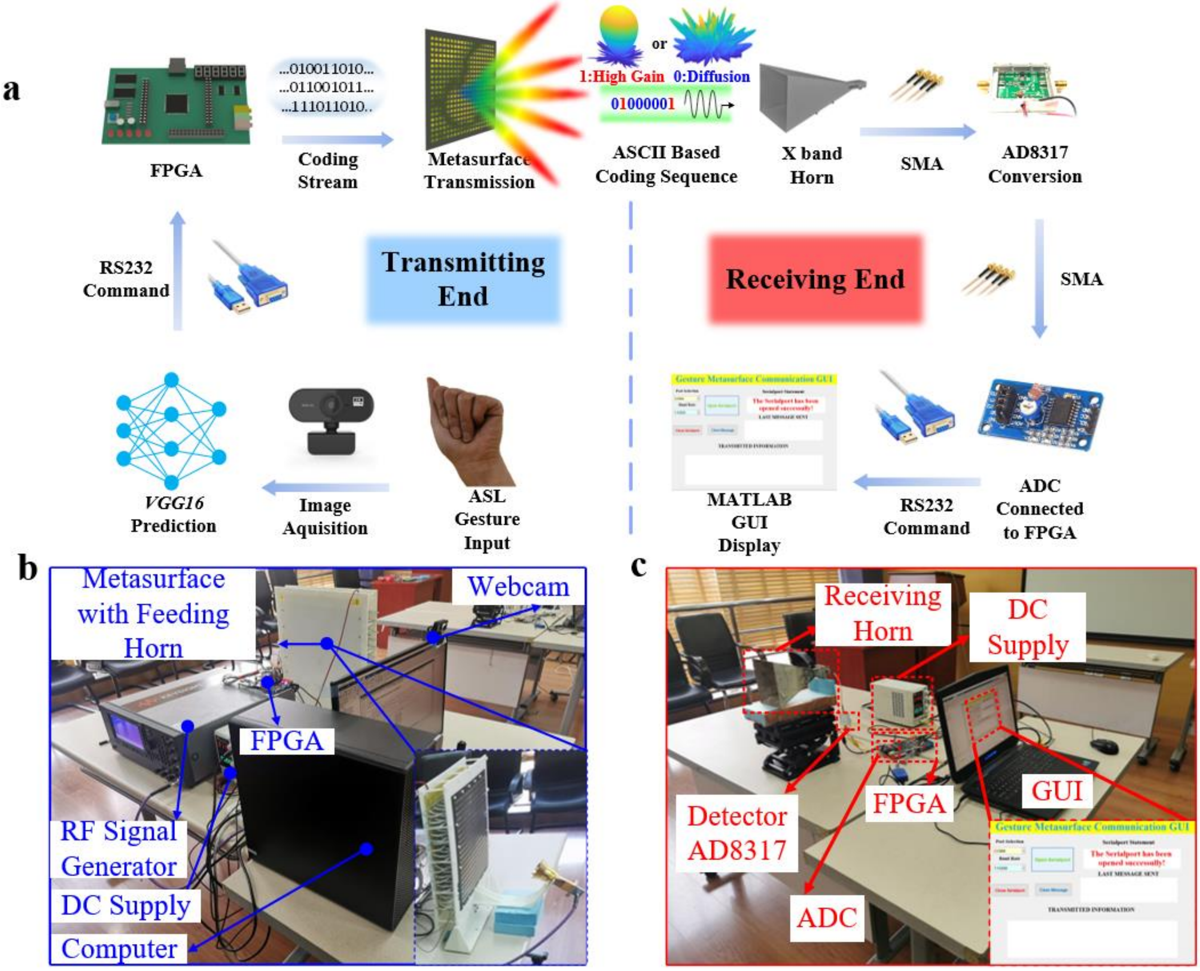

Figure S9. Workflow of wireless communication and experimental setup for transmitting end and receiving end. a. Operation process of GMI system in wireless communication. b. Equipment arrangement at transmitting end. c. Equipment arrangement at receiving end.

S6.2. Experimental setup for Wireless Communication

Figure S9b shows the detailed experimental setup of the transmitting end, which is composed of an RF signal generator (Keysight E8267D) for feeding the horn antenna, a DC power supply for

driving the control circuit, a webcam for capturing the gesture images, and a computer for data processing. Figure S9c shows the detailed experimental setup of the receiving end, which is composed of a horn antenna for receiving signals, a detector (AD8317) for converting the RF signal to a DC analog signal, another DC power supply for driving the detector, a FPGA with an ADC (AD9280 chip) for signal conversion, and a notebook computer for displaying the text message.

It should be noted that the distance between metasurface and receiving horn can be further increased for far-field (more than 6 meters) communication in theory. However, if a longer communication distance is required, a power amplifier (PA) and a low noise amplifier (LNA) should be added before feeding horn and after receiving horn respectively to against the space attenuation.

Section S7. Phase compensation of different-type beams in beam manipulation

For all kinds of beams, such as pencil-like beam, dual beam, OAM beam, diffusion beam and Bessel beam in this work, the same phase compensation is firstly required for metasurface to eliminate the phase difference caused by the feeding horn antenna. Assuming that the distance between the ith element and phase center of the feeding horn antenna is d_i, the spatial phase delay can be calculated as

$$\phi_{i\_spd} = -k_0 \cdot d_i \tag{S1}$$

where k_0 is the wave number in free space.

The additional required phase compensations for pencil-like beam, multi-beam, OAM beam, diffusion beam and Bessel beam will be further discussed in the following text, respectively.

Pencil-like beam. Based on reflectarray antenna theory, the required phase compensation at different positions (x_i, y_i) of metasurface for a pencil-like beam with a deflection angle of (θ, φ) can be given by

$$\phi_{i\_pencil} = -\phi_{i\_spd} + k_0 \cdot \left(x_i \sin\theta \cos\varphi + y_i \sin\theta \sin\varphi \right) \tag{S2}$$

where θ and φ represent the elevation angle and azimuth angle in the spherical coordinate system, respectively.

Bessel beam. For the Bessel beam, the phase distribution of metasurface mainly depends on the

designed range of depth-of-field vector $\boldsymbol{L}$, the elevation angle θ and the azimuth angle φ. The main purpose is to find a conical surface phase distribution that satisfies the parameter mentioned above. Assume that the element lies on the corner of metasurface is R away from the central position (any one of four), the convergence angle δ (angle between $\boldsymbol{S}$ and $\boldsymbol{L}$) and the angle γ (angle between L and generatrix of phase conical surface) should be determined first through $\boldsymbol{L}$, $\boldsymbol{R}$, and (θ, φ). S is the vector that equals to $\boldsymbol{L}$+$\boldsymbol{R}$. Based on the Cosine Law

$$\delta = \cos^{-1}\left(\frac{\vec{L}^2 + \vec{S}^2 - \vec{R}^2}{2\left|\vec{L}\right|\left|\vec{S}\right|}\right), \text{ where } \left|\vec{S}\right| = \sqrt{(\vec{L}^2 + \vec{R}^2 - 2\left|\vec{L}\right|\left|\vec{R}\right|\cos(\frac{\pi}{2}-\theta))} \qquad \text{(S3)}$$

$$\gamma = \frac{\pi}{2} - \delta \qquad \text{(S4)}$$

where γ is the angle beween the direction (θ, φ) and generatrix. Then, the focal spot vector $\boldsymbol{l_i}$ is deduced, where the focal spot is contributed by ith element. According to the property of similar triangles

$$\left|\vec{l}_i\right| = \frac{\left|\vec{r}_i\right|}{\left|\vec{R}\right|} \text{ , where } \left|\vec{r}_i\right| = \sqrt{(x_i^2 + y_i^2)} \qquad \text{(S5)}$$

where $\boldsymbol{r_i}$ represents the ith element position vector. One step further, we can calculate the distance s_i which equals to |$\boldsymbol{l_i}$-$\boldsymbol{r_i}$|, then, the angle between direction (θ, φ) and $\boldsymbol{r_i}$, that is α_i. Once α_i is determined, ζ_i (the angle between $\boldsymbol{r_i}$ and the projection of $\boldsymbol{r_i}$ on phase conical surface) can be calculated.

$$s_i = \sqrt{\left(\left|\vec{l}_i\right|\sin(\frac{\pi}{2}-\theta)\right)^2 + \left(\left|\vec{l}_i\right|\cos(\frac{\pi}{2}-\theta)\cos\varphi - x_i\right)^2 + \left(\left|\vec{l}_i\right|\cos(\frac{\pi}{2}-\theta)\sin\varphi - y_i\right)^2} \qquad \text{(S6)}$$

$$\alpha_i = \cos^{-1}\left(\frac{\vec{l}_i^2 - \vec{r}_i^2 - s_i^2}{2\left|\vec{r}_i\right|\left|\vec{l}_i\right|}\right) \qquad \text{(S7)}$$

$$\zeta_i = \alpha_i - \gamma \qquad \text{(S8)}$$

Finally, the total compensation phase is

$$\phi_{i\_Bes} = -\phi_{i\_spd} + k_0\left|\vec{r}_i\right|\sin\left(\zeta_i\right) \qquad \text{(S9)}$$

In this scheme, elevation angles θ=0, ±10°, ±20°, ±30°, ±40°, ±50°, ±60°, φ=0 and |$\boldsymbol{L}$|=400 mm are

selected as demonstration in real-time beam manipulation.

Multiple beams. For beams pointing at directions (θ_1, φ_1), (θ_2, φ_2),…(θ_i, φ_i)… (θ_n, φ_n), according to Eq. (2), we can get their phase distributions ϕ_1, ϕ_2,… ϕ_i,… ϕ_n, respectively, and then the complex field distribution $\boldsymbol{\sigma_i}$ for *i*th beam can be written as

$$\boldsymbol{\sigma}_i = e^{j\phi_i} \tag{S10}$$

Based on the field superposition principle, the total phase distribution of metasurface for the multi-beam radiation can be calculated by

$$\phi_{multi} = \angle \sum_{i=1}^{i=n} \boldsymbol{\sigma}_i \tag{S11}$$

Here, a dual-beam point at (-30°, 0) and (30°, 0) is shown as the demonstration.

Diffusion beam. In order to achieve the diffusion beam, the random phase distribution is required for the metasurface. In this scheme, the phase of *i*th element of metasurface is set as

$$\phi_{i\_diff} = \phi_{i\_spd} + randi([0,3]) \cdot \frac{\pi}{2} \tag{S12}$$

where *randi*([0,3]) means the generation of a random integer from 0 to 3.

OAM beam. For general form of OAM beam, the required phase distribution of metasurface can be calculated by

$$\phi^{l}_{i\_OAM} = -\phi_{i\_spd} + k_0 \cdot \left(x_i \sin\theta \cos\varphi + y_i \sin\theta \sin\varphi\right) + l \cdot \arctan\left(\frac{y_i}{x_i}\right) \tag{S13}$$

where l indicates the topological charge. In this scheme, we only considered $\theta=\varphi=0°$, and $l=\pm3, \pm2, \pm1$ for simplicity.

Section S8. OOK modulation and demodulation in wireless communication

For a wireless communication system, the distance between the receiving horn antenna and metasurface is 2 m, when the metasurface works on high gain pattern and low gain pattern, the signals are received by the receiving horn antenna, and the outputs of the AD8317 module are about 0.55 V and 1.58 V, respectively. Therefore, we set the criteria value in Analog Digital Converter

(ADC) as 1V, that is, when the metasurface operates on high gain pattern, the input voltage of ADC is 0.55V (less than 1V criteria value), the ADC outputs digital code “1”, otherwise, it outputs digital code “0”. Because most of the transmitted gestures are defined based on the ASCII code, the 8-bit binary numbers to the corresponding characters can be directly converted. However, for the other self-defined gestures, one-to-one correspondence between the 8-bit binary codes and meaning of gestures needs to be first established. The receiving end just needs to extract the correct 8-bit sequence, and then the sending message can be recovered.

In the receiving end, the horn keeps sampling receiving signals. In the ASCII code table, each symbol starts with 0 (for example, 01000001 represents A), and no symbol starts with 1, indicating that 0 can be treated as the check code. If the metasurface keeps high gain (code 1) while the system is waiting for the orders, when a symbol is sent by the metasurface (OOK modulation), once the code 0 is firstly shifted as the start bit, we can accurately start to record the symbol we sent. However, if a diffusion pattern (code 0) is set as the default mode while the system is waiting for orders, it is relatively complex to recover the information, as shown in Figure S10.

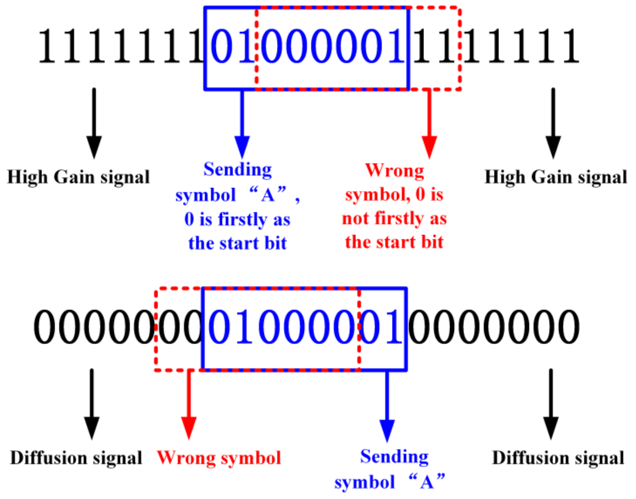

Figure S10. The illustration for choosing high gain as the default mode when the webcam is waiting for orders.

Section S9. Response time for the system

The response speed of the system itself, whether operating in beam manipulation or communication are in millisecond level. For beam manipulation mode, the skin-color detection region will take about 9 ms to make a judgment. Once the beam direction is determined, the beam direction command will be sent to the FPGA. This process is determined by the baud rate of the serial port. In our case, it is 115200 bit/s. For an 8-bit command, this costs about 7 us. After that, the

corresponding pattern will be loaded to the metasurface which costs about 25.6 us. The total time consumption for beam manipulation is less than 10 ms.

For the communication mode, each gesture represents one alphabet, a simple OOK is applied, the theoretical recovering time for one alphabet is 8 (sampling count, 8-bit ASCII code)$\times$2.048 ms(sampling time interval) = 16.384 ms. Limited by the experimental computer (Intel i7 9700, 32GB RAM), the response time of the trained network is about 70 ms. The recovering time in the matlab program is 3 ms. In theory, the response speed for each symbol should be less than 90 ms (including command-send time consumption).

In actual, the total response speed is mainly influenced by the gesture capture process. Considering the operator is unfamiliar with sign languages, we set the camera sampling time to 3 seconds, which can be observed in the supplementary videos, so the total time for each symbol transmitting should be about 3.09 seconds in this design. However, this sampling time can be dramatically reduced by practicing sign languages, and thereby greatly improving the whole response speed of system.

Appendix SI: Gestures definition for beam manipulation

VGG16 Network1			
BM Gestures (*ASL Standard*)			
Sequence Number	Gesture Image	Meaning	Mode
0	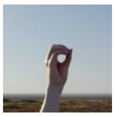	Number “0”	
1	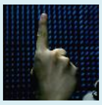	Number “1”	High Gain
2	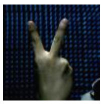	Number “2”	Dual Beam
3	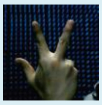	Number “3”	OAM

4	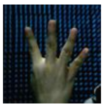	Number “4”	Diffusion
5	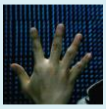	Number “5”	Bessel Beam
6	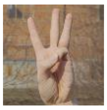	Number “6”	
7	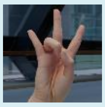	Number “7”	
8	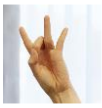	Number “8”	
9	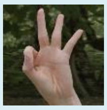	Number “9”	
10	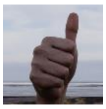	Number “10”	
11	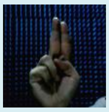	Plus	OAM +1
12	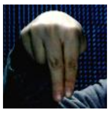	Minus	OAM -1

Appendix SII: Gestures definition for Wireless Communication

VGG16 Network2				
WC Gestures (*ASL Standard Alphabet*)				
Sequence Number	Gesture Image	Meaning	ASCII	RS232 Command
1	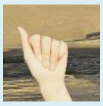	Key “A”	01000001	00000001
2	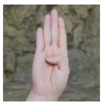	Key “B”	01000010	00000010

3	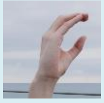	Key “C”	01000011	00000011
4	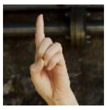	Key “D”	01000100	00000100
5	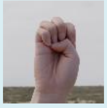	Key “E”	01000101	00000101
6	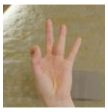	Key “F”	01000110	00000110
7	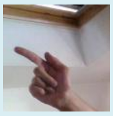	Key “G”	01000111	00000111
8	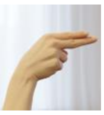	Key “H”	01001000	00001000
9	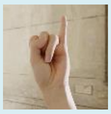	Key “I”	01001001	00001001
10	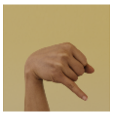	Key “J”	01001010	00001010
11	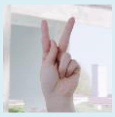	Key “K”	01001011	00001011
12	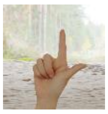	Key “L”	01001100	00001100
13	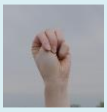	Key “M”	01001101	00001101
14	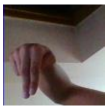	Key “N”	01001110	00001110
15	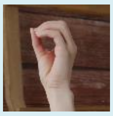	Key “O”	01001111	00001111
16	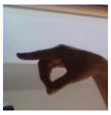	Key “P”	01010000	00010000
17	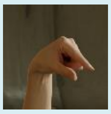	Key “Q”	01010001	00010001

18	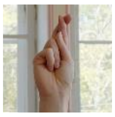	Key “R”	01010010	00010010
19	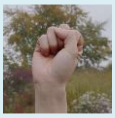	Key “S”	01010011	00010011
20	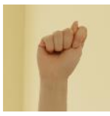	Key “T”	01010100	00010100
21	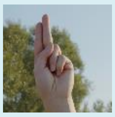	Key “U”	01010101	00010101
22	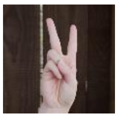	Key “V”	01010110	00010110
23	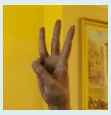	Key “W”	01010111	00010111
24	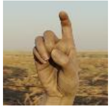	Key “X”	01011000	00011000
25	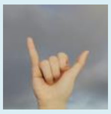	Key “Y”	01011001	00011001
26	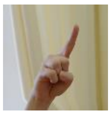	Key “Z”	01011010	00011010

WC Gestures (*Partial Self-defined Punctuation and Function Keys*)

Sequence Number	Gesture Image	Meaning	ASCII	RS232 Command
27	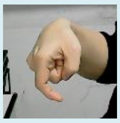	Comma	00101100	00011011
28	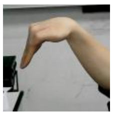	Backspace	00001000	00011100
29	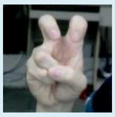	Double Quotation	00100010	00011101
30	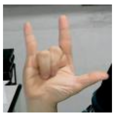	Exclamation Mark	00100001	00011110

31	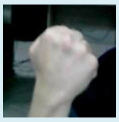	Full Stop	00101110	00011111
32	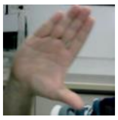	Left Bracket	00101000	00100000
33	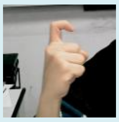	Question Mark	00111111	00100001
34	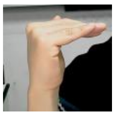	Return	00001101	00100010
35	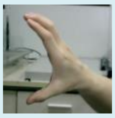	Right Bracket	00101001	00100011
36	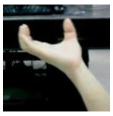	Space	00100000	00100100

WC Gestures (*Self-defined Kaomoji*)

Sequence Number	Gesture Image	Meaning	ASCII	RS232 Command
37	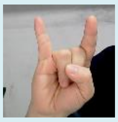	Happy !(^o^)!	00001001	00100101
38	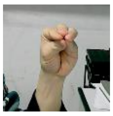	Kiss o(*￣3￣)o	00001100	00100110
39	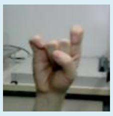	Smile (^ω^)	00001011	00100111
40	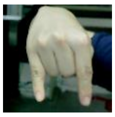	Unhappy (*￣︿￣)	00001010	00101000

References

[S1] Nexperia,74HC595D Datasheet https://assets.nexperia.com/documents/data-sheet/74HC_HCT595.pdf, **2021**.
